# Solutions to Cases Described on Page 238

**Published:** 1908-06-06

**Authors:** 


### Cases for Diagnosis.

### SOLUTIONS TO CASES DESCRIBED ON PAGE 238.

*Case* 1.—The diagnosis was thought to be either acute intestinal obstruction by a band or by a growth, or else acute hæmorrhagic pancreatitis. In view of the presence of jaundice and the absence of visible peristalsis, the latter seemed to be the more likely of the two.

It was decided to give a large soap-and-water enema at once to try and bring about an evacuation of the bowels; and, if no such evacuation resulted, it was considered advisable to proceed at once to laparotomy for the relief of any obstruction there might be.

The soap enema was given, retained for a short while, and then returned without any result either solid or gaseous. The pulse almost immediately after failed to such a degree that it was deemed advisable not to operate unless it should improve; it remained extremely rapid, however, and almost impalpable, and it was clear that an anæsthetic was out of the question. She was moribund, and died about an hour later.

At the autopsy the cause of the trouble was found to be mesenteric thrombosis, apparently spontaneous. There was a piece of dark purple, almost gangrenous bowel just below the duodeno-jejunal flexure; the duodenum itself was very dilated, and so was the jejunum for about three feet below the diseased part. There was no ulceration within the bowel, but the mucosa was injected and inflamed, with numerous submucous and subserous extravasations of blood. The inflammation had spread up the duodenum far enough to affect the biliary papilla and cause jaundice. The branches of the mesenteric artery and vein were both thrombosed in the region of the affected area of jejunum. There was much local plastic peritonitis, with early acute inflammation of the rest of the peritoneum, and a very small quantity of free fluid. The rest of the alimentary canal was quite healthy. The heart, lungs, liver, gall-bladder, and all the other organs were natural except the kidneys which, though not diminished in size, had thickened adherent capsules, granular surfaces from which several retention cysts projected, and diminished width of cortex.

*Case* 2.—The provisional diagnosis was that the patient had had mild attacks of appendicular pain at previous times, and had now been attacked by a more serious appenditicis which had first led to local inflammation in the right iliac fossa, and then to widespread, if not absolutely general peritonitis. With the rising pulse-rate and the persistent vomiting it did not seem justifiable to postpone laparotomy, which was performed almost at once. The incision was in the middle line below the umbilicus to begin with; no peritonitis was found, and to the surprise of all the vermiform appendix turned out to be absolutely healthy. The incision was enlarged in the expectation of finding the source of the symptoms elsewhere. The fullness that had been noticed in the right iliac fossa was found to be a somewhat enlarged and very movable kidney, but nothing very wrong with it could be found on palpation. The stomach and duodenum were examined for ulcer, with possibly a local perforation, but without success. The whole of the intestine, the gall-bladder, the pelvic viscera, were also examined carefully, but no gross lesion could be found, and the patient was taken back to bed with the mystery of her disease still unsolved. The only point noted was that the small intestine was collapsed and empty while the large intestine was distended with gas.

The operation was on June 3; the patient rallied well, but was very sick. The vomiting was partly due to the anæsthetic, partly to the persistence of her original trouble, and partly to the extensive manipulation of her viscera, with dragging on the solar plexus in the process of searching the abdomen. On June 6 the vomiting was getting worse rather than better, and the pulse-rate remained high; the stomach was washed out. This relieved the trouble for a time, but by June 8 the vomiting, retching, and abdominal distension had become terrible again; about mid-day an immense flood of dark brown fluid regurgitated from the stomach through her mouth and nostrils, the face became extremely cyanosed in a moment, and the patient died.

The diagnosis was still as obscure as before, and the findings at the autopsy were quite unexpected. The immediate cause of death was an extraordinary degree of acute dilatation of the stomach; this was in no way connected with the original symptoms, presumably, but was rather due to paralytic distension of the viscus from the traction upon the solar plexus that seemed unavoidable at the operation. The upper part of the distended stomach reached as high as the third rib, and the lower part of the organ was in the pelvis; it looked like the bag of a bagpipes, and was full of gas and many pints of the same kind of dark brown fluid as had escaped from the mouth just before death. The rest of the intestinal tract was healthy; the uterus, vagina, ovaries, and Fallopian tubes were all natural. There was nothing materially wrong with any other organ except the right kidney and ureter. The left kidney was natural, but the right weighed 9½ oz. Its surface was granular and irregular, its capsule adherent, its cortex thinned, its medulla blotchy-looking, and its pelvis considerably dilated. Firmly imbedded in a cavity formed in the apex of one of the pyramids was a rough non-facetted stone about ½ in. in diameter, together with a number of small stones about the size of No. 6 shot. Before opening the kidney, it was carefully palpated to see if any stone could be felt inside it, but none could be detected; on closing the kidney up again, it was found that only in one position of the finger and thumb could the stone be felt, showing how easy it is to overlook a stone in palpating the kidney even in the post-mortem room, and therefore how very difficult it must be to be sure that no stone is present when the organ is palpated by the surgeon in the living subject. The right ureter was a little dilated, and the lowest 1½ in. were lined by adherent yellow membrane, removal of which showed excoriation and very acute inflammation of the mucosa and submucosa at this spot. There was no stone in the bladder. Sections of the right kidney under the microscope showed tracts of small round celled infiltration, the result of recent acute ascending nephritis, and in addition a considerable degree of old interstitial fibrosis.

It was clear that the symptoms of appendicitis in this case had been simulated by *acute ureteritis* due to excoriation by a small calculus. At the time the patient's condition seemed too bad for cystoscopy to be considered; yet looking back at the case it is clear that the slight hæmaturia attracted insufficient attention, and was too lightly attributed to the expected menstruation. It is easy to be wise after the event, but had one waited, or had one thought of using the cystoscope, it is probable that no laparotomy would have been done. Death, however, would in all probability have been postponed only for a short while, because the acute ascending nephritis was already marked; but the case is instructive; and if there are two side-issues upon which it enables one to lay emphasis they are:—

1. The difficulty of being certain that a kidney contains no stone, so hard is it to feel a small calculus even when the kidney is in one's hands.

2. The danger of acute dilatation of the stomach after prolonged manipulations in cases of abdominal section involving traction on the mesentery.

